# A Continent-Wide Migratory Divide in North American Breeding Barn Swallows (*Hirundo rustica*)

**DOI:** 10.1371/journal.pone.0129340

**Published:** 2015-06-11

**Authors:** Keith A. Hobson, Kevin J. Kardynal, Steven L. Van Wilgenburg, Gretchen Albrecht, Antonio Salvadori, Michael D. Cadman, Felix Liechti, James W. Fox

**Affiliations:** 1 Science and Technology Branch, Environment Canada, Saskatoon, Saskatchewan, Canada; 2 Woodland Park Zoo, Seattle, Washington, United States of America; 3 17 Colborn St., Guelph, Ontario, Canada; 4 Canadian Wildlife Service, Environment Canada, Burlington, Ontario, Canada; 5 Swiss Ornithological Institute, Sempach, Switzerland; 6 Migrate Technology, Cambridge, United Kingdom; University of Regina, CANADA

## Abstract

Populations of most North American aerial insectivores have undergone steep population declines over the past 40 years but the relative importance of factors operating on breeding, wintering, or stopover sites remains unknown. We used archival light-level geolocators to track the phenology, movements and winter locations of barn swallows (*Hirdundo rustica*; n = 27) from populations across North America to determine their migratory connectivity. We identified an east-west continental migratory divide for barn swallows with birds from western regions (Washington State, USA (n = 8) and Saskatchewan, Canada (n = 5)) traveling shorter distances to wintering areas ranging from Oregon to northern Colombia than eastern populations (Ontario (n = 3) and New Brunswick (n = 10), Canada) which wintered in South America south of the Amazon basin. A single swallow from a stable population in Alabama shared a similar migration route to eastern barn swallows but wintered farther north in northeast Brazil indicating a potential leap frog pattern migratory among eastern birds. Six of 9 (67%) birds from the two eastern populations and Alabama underwent a loop migration west of fall migration routes including around the Gulf of Mexico travelling a mean of 2,224 km and 722 km longer on spring migration, respectively. Longer migration distances, including the requirement to cross the Caribbean Sea and Gulf of Mexico and subsequent shorter sedentary wintering periods, may exacerbate declines for populations breeding in northeastern North America.

## Introduction

Conserving migratory birds is challenging in part because they spend their annual cycle at various (often poorly known) locations that can be separated by thousands of kilometers. By establishing links between breeding, wintering and stopover locations for populations of interest, it is possible to test hypotheses of where key factors may be operating to reduce productivity and survivorship [1]. For example, interactions between events occurring on the breeding grounds and carry-over effects from wintering and migration can have major influences on productivity and survivorship [2, 3]. Survivorship can also be influenced differentially throughout the annual cycle but it is suspected that most annual mortality among adults occurs during the non-breeding period including migration [4–6]. At continental scales, it can thus be useful to compare population trajectories among populations that differ in their migratory routes and overall migratory connectivity in order to evaluate likely biotic (e.g. habitat quality, prey availability, predation) and abiotic (e.g. climate cycles, weather) processes occurring at different times and locations. Separate breeding populations sharing common wintering areas and migration routes would be expected to be affected similarly by processes occurring off the breeding grounds compared to those breeding populations that show weak similarity [7, 8]. Delineating broad patterns of migratory connectivity at continental scales can be a useful first step in evaluating causes of differential population trends among locations. Unfortunately, for most migratory species, we typically lack basic information on migratory connectivity in order to test such hypotheses.

In North America, aerial insectivorous birds including the barn swallow (*Hirundo rustica*), have undergone steep declines since the 1970s [9]. On the breeding grounds, potential causes for their decline likely include changing land-use practices, particularly agricultural intensification and increased pesticide use [9, 10]; events which may also be encountered on the wintering grounds [11, 12]. Worsening climate or weather patterns on breeding, wintering or stopover sites might also play a role. Indeed, in a recent extensive analysis of population trends of five aerial insectivore species breeding in North America, Michel, Smith [13] determined that climate cycles account for up to 47% of variation in population trends regionally on the breeding grounds and these factors can operate differently for populations in different regions across the continent. In the western hemisphere, the majority of barn swallows breed in North and Central America and winter from southern Mexico through most of South America [14]. Migration routes are overland through Central America as well as across the Gulf of Mexico and the Caribbean Islands but little is known about population-specific routes or linkages between breeding and wintering locations. Using stable isotopes to assess wintering provenance, García-Pérez, Hobson [15] postulated a longitudinal structure in migratory connectivity with birds breeding in western and northern North America migrating to western South America and eastern and southern breeding birds migrating to northeast regions of South America. However, that study assumed the only possible wintering sites to be in South America.

The recent miniaturization of archival light-level geolocators (hereafter, geolocators) has revolutionized the tracking of small passerines through the annual cycle and has advanced our understanding of migration routes, wintering areas, stopover sites and phenology of many species [16]. Geolocators store light-level data that is used to provide an estimate of latitude based on day length and longitude based on time of mid-day [17]. Errors for latitude (± 300–365 km) positions are typically higher than for longitude (± 66–150 km) due to the influence of shading (e.g. variable cloud cover, habitat) on light levels [18–20]; however, geolocation remains one of the most accurate tracking methods for small passerines. We used geolocators to determine migration routes and wintering areas of multiple barn swallow breeding populations with differing population trends from across North America. The objectives of this study were to: 1) assess the strength of migratory connectivity of barn swallow populations breeding in different parts of North America; and 2) identify non-breeding sites, migration routes and overall non-breeding season phenology which could provide insights into possible factors influencing regional population trends. Based on results from an earlier isotope study [21], we define barn swallows nesting in Washington State and Saskatchewan as western populations and those nesting in Ontario and New Brunswick as eastern populations.

## Methods

Barn swallows were captured during the breeding season (May–July) from five populations across a broad geographic extent (Washington State and Alabama, USA; Saskatchewan, Ontario, New Brunswick, Canada; Table 1) using mist-nets. Individuals were then sexed by determining the presence of a cloacal protuberance (male) or brood patch (female), weighed (to 0.1 g) and fit with a uniquely numbered U.S. Geological Survey metal leg band. Birds ≥17 g (i.e. geolocator plus harness weight ≤5% of total body mass) were fit with geolocators using a leg-loop backpack harness [22] constructed from 1 mm silicone string sized appropriately for each bird (i.e. Naef-Daenzer [23] formula). We used Swiss Ornithological Institute (SOI) GDL 3.0 geolocators (Sempach, Switzerland; 0.65 ± 0.02 g) in 2011 and 2012 and Migrate Technology (Intigeo-P55B1-7 Cambridge, UK; 0.65 ± 0.1 g) in 2013. This study was carried out in accordance with the recommendations by the Canadian Council of Animal Care and the protocol was approved by Environment Canada’s Animal Care Committee (Protocol ID # EC-PN-11-030).

**Table 1:** Summary information, wintering distance and estimated fall and spring migration distances for barn swallows fit with archival light-level geolocators from five breeding populations, 2011–2013.

Breeding Population	Capture Location		Geolocators		Mean (± 1SD) within-population wintering distance (km; orthodromic) apart	Mean (± 1SD) estimated travel distance (km) to wintering grounds	Mean (± 1SD) estimated travel distance to breeding grounds (km)
	Latitude	Longitude	Deployed	Recaptured			
Washington State	47.67	-122.35	40	9	2,093 (± 1,401)	4,805 (± 2,038); n = 8	4,528 (± 2,558); n = 5
Saskatchewan	53.59	-106.05	33	5	948 (± 603)	7,781 (± 949); n = 5	7,526 (± 758); n = 4
Ontario	43.74	-80.15	13	3	1,488 (± 663)	8,807 (±1,393); n = 3	10,133 (± 604); n = 3
New Brunswick	45.93	-65.26	16	10	1,793 (± 867)	10,423 (± 905); n = 10	11,288 (± 2,004); n = 5
Alabama	32.57	-85.36	18	5	NA	6,198; n = 1	6,918; n = 1

We derived position estimates from light-level geolocation data using the GeoLight package [24] in the program R v. 2.15.2 [25] and Migrate Technology’s IntiProc V1.02 user interface to the GeoLight package for IOS and Migrate Technology geolocators, respectively. Man-made structures (e.g. buildings, culverts) used by barn swallows for nesting and roosting [14] have a large impact on position estimates through shading and so units were calibrated for 10 days after birds were assumed to have completed nesting (by inspection of light level curves) and remained on their breeding territory but would not be in shaded structures (e.g. barns) for extended time periods. We used the same sun elevation angle for individual birds for all non-breeding periods because accuracy of position estimates during this period (i.e. migration, wintering) should be relatively unaffected by shading when barn swallows only use open habitats (e.g. marshes, crops [14]). The mean calibrated sun elevation angle for birds (*n* = 27) ranged from -0.7° to -5.5° (mean = -3.4 ± 1.3°). Inaccuracy (ignoring direction) in raw geolocations during the calibration period averaged 246 ± 240 km latitude and 120 ± 123 km longitude.

To increase the accuracy of positions from raw geolocations and assist with data interpretation, we applied a state-space Kalman filter model which estimates errors, movement parameters, and ‘most probable tracks’ [26, 27] with the package kftrack [28] in R v2.15.2 [25]. The Kalman filter model assumes that raw geolocations of a marked organism are representative of true positions with some measurement errors and predicts movement via a biased random walk [26, 29] where the estimated location at time t+1 after an initial known location at time t is Gaussian with associated error (i.e. mean and variance [30]). The Kalman filter recursively estimates the locations of the tagged individual along with the variance components of the estimated position at each time step. Residuals of the difference between the estimated random walk position and the raw geolocation position are used to calculate a likelihood function. A ‘most probable position’ is then computed as a trade-off between the random walk position and the position estimated by the tag (input data) based on the relative variance of the two estimates. We set the diffusion component (variability in movement) of the model to the estimated maximum flight distance of barn swallows during migration in North America (194 km [14]). We calculated the systematic error in the longitude and latitude from differences in raw positions for the same 10-day period used to calibrate sun elevation angles and we used the “uniform” variance structure (i.e. equal variance in location assumed for all observations). These input parameters are used to provide initial values but do not influence resulting parameter estimates [26].

Breeding ground departure and arrival dates were initially estimated by visually assessing raw light data for obvious changes in light levels associated with birds visiting shaded nesting structures similar to Liechti, Scandolara [31]. In that study, ‘clean’ light level curves were associated with the end of breeding and the onset of migration [31]. However, inspection of our raw geolocation data indicated that it was likely some of the birds remained close to their nesting sites but were not roosting in the nesting structure prior to migration. Therefore, using the results from the state-space Kalman filter models, large (≥ 150 km) consistent (e.g., south) directional movements away from the breeding grounds for two or more days were used as determinants of departure from the breeding grounds including during the equinox when only longitude could be used [32]. Start and end dates of the wintering period were first estimated using the changeLight function in the R GeoLight package [24] which iteratively searches for breakpoints in the light data to determine potential residency and migration periods. This method was useful in providing preliminary estimates of wintering dates; however, it often calculated multiple residency periods within a short timeframe and distances within the range of geolocation error that could not be reliably considered separate wintering areas. Thus, similar to our estimates of breeding ground departure and arrival dates, we calculated mean positions for sedentary winter locations when no large (≥ 150 km) unidirectional movements were detected for two or more days for individual birds. We considered the wintering area as the general location in which a bird remained for the longest period during the non-breeding season given the above criteria and classed all other stationary periods as potential stopovers. Directional standard deviational ellipses were then derived from the points (i.e. daily location estimates) considered part of the wintering location to represent positional error during that period.

Estimation of latitude is unreliable during the spring and fall equinoxes when length of day and night are approximately equal for two weeks. Positions during the equinoxes can be estimated using the state-space model; however, some locations during these periods were suspect (e.g. several hundred to thousands of kilometers from previous position; over the ocean) therefore we used longitude data only from September 8—October 5, 2013 and March 6—April 4, 2014 and geographical features (e.g. Caribbean Islands) to estimate locations during this period. Results were evaluated for model convergence and other parameter estimates (e.g., diffusivity, latitudinal error) in addition to comparison of the position estimates from the state-space model with raw geolocations.

### Statistical Analysis

We tested for differences in multiple response variables (e.g. timing of arrival on wintering and breeding grounds, estimated distance travelled; see Table 2) using general linear models (GLM) with sampling population (e.g., Washington State, New Brunswick) and sex included as explanatory variables. We did not analyze any interactions between variables to avoid over-parameterization with our small sample size. Preliminary models included ‘year’ as an explanatory factor to test for its effect on the data and it was only significant for length of time at wintering grounds. However, this variable was removed from subsequent analyses and all of the data was grouped across years because we could not accurately interpret the effect of year due to a lack of replicates for individuals and location-specific populations in multiple years (i.e. Ontario samples were all from one year). We also ran a GLM to determine if there were differences in the orthodromic (Vincenty great circle method [33]) distance between breeding and wintering grounds because estimates of travel routes could possibly have large errors due to geolocation inaccuracies and an inability to produce geolocations during the equinox. Post-hoc comparisons with a Bonferroni correction were conducted to determine pair-wise effects for each test. We also used multivariate analysis of variance (MANOVA) on mean wintering ground coordinates (latitude and longitude) to test differences between sexes and populations. Ten of the geolocators stopped recording after the birds reached the presumed wintering grounds or during spring migration. One geolocator with useable data was recovered from barn swallows in Alabama but was not included in the analyses. We considered variables to be significant at P < 0.05.

**Table 2:** Results from linear models used to describe differences in breeding and wintering ground phenology, wintering locations and migration routes for male and female barn swallows fit with archival light-level geolocators (n = 27) from four populations in North America, 2011–2013.

Variable	Sex				Population			
	F	DF	*p*	adj. R^2^	F	DF	*p*	adj. R^2^
Departure date from breeding grounds	3.53	1,23	0.07	0.10	**6.35**	**3,22**	**<0.005**	**0.39**
Predicted travel distance to wintering grounds	2.32	1,23	0.14	0.05	**23.76**	**3,22**	**< 0.001**	**0.73**
Orthodromic distance between breeding and wintering grounds	1.41	1,23	0.25	0.02	**16.66**	**3,22**	**< 0.001**	**0.65**
Number of days to reach wintering grounds	0.19	1,23	0.66	-0.03	**5.25**	**3,22**	**<0.01**	**0.34**
Winter longitude	2.09	1,23	0.16	0.04	**33.61**	**3,22**	**< 0.001**	**0.80**
Winter latitude	2.22	1,23	0.15	0.05	**38.06**	**3,22**	**< 0.001**	**0.82**
Arrival date on wintering grounds	1.66	1,23	0.21	0.03	**9.24**	**3,22**	**< 0.001**	**0.50**
Duration (d) at wintering location	2.88	1,18	0.11	0.04	0.74	3,16	0.54	-0.04
Departure date from wintering grounds	0.23	1,18	0.63	-0.04	**19.51**	**3,14**	**> 0.001**	**0.77**
Predicted travel distance to breeding grounds	3.69	1,15	0.26	0.14	**12.42**	**3,13**	**< 0.001**	**0.68**
Arrival date to breeding grounds	0.36	1,13	0.56	-0.05	**4.79**	**3,11**	**< 0.05**	**0.49**
Days to reach breeding grounds	1.40	1,13	0.22	0.03	**12.15**	**3,11**	**< 0.001**	**0.71**

Statistically significant (P < 0.05) effects are shown in bold.

## Results

### Return Rates of Birds with Geolocators

We recovered 32 geolocators from returning birds of which four of the SOI model geolocators failed and one of the Migrate Technology geolocators collected data for approximately two weeks. Therefore, we were able to retrieve data from 27 working geolocators (13 females, 13 males, one unknown sex) of which 17 had data for one full cycle (i.e. deployment to recapture on the breeding grounds in the subsequent year; Table 1). We could not estimate breeding ground arrival date for three birds for which geolocator battery failed near the breeding grounds. Migration travel distances for these swallows were estimated by extending the travel route from the last reliable geolocation to the breeding site. Our return rate of 26.7% across populations is similar to or lower than those for a study of barn swallows without geolocators at three of the same locations (Ontario = 25.9%; New Brunswick = 43%; Washington State = 35.0%) [15]; however, our sampling effort was substantially lower for some populations (i.e. we sampled for several hours versus months at most capture locations).

### Fall Migration Timing and Routes

**Fig 1:**
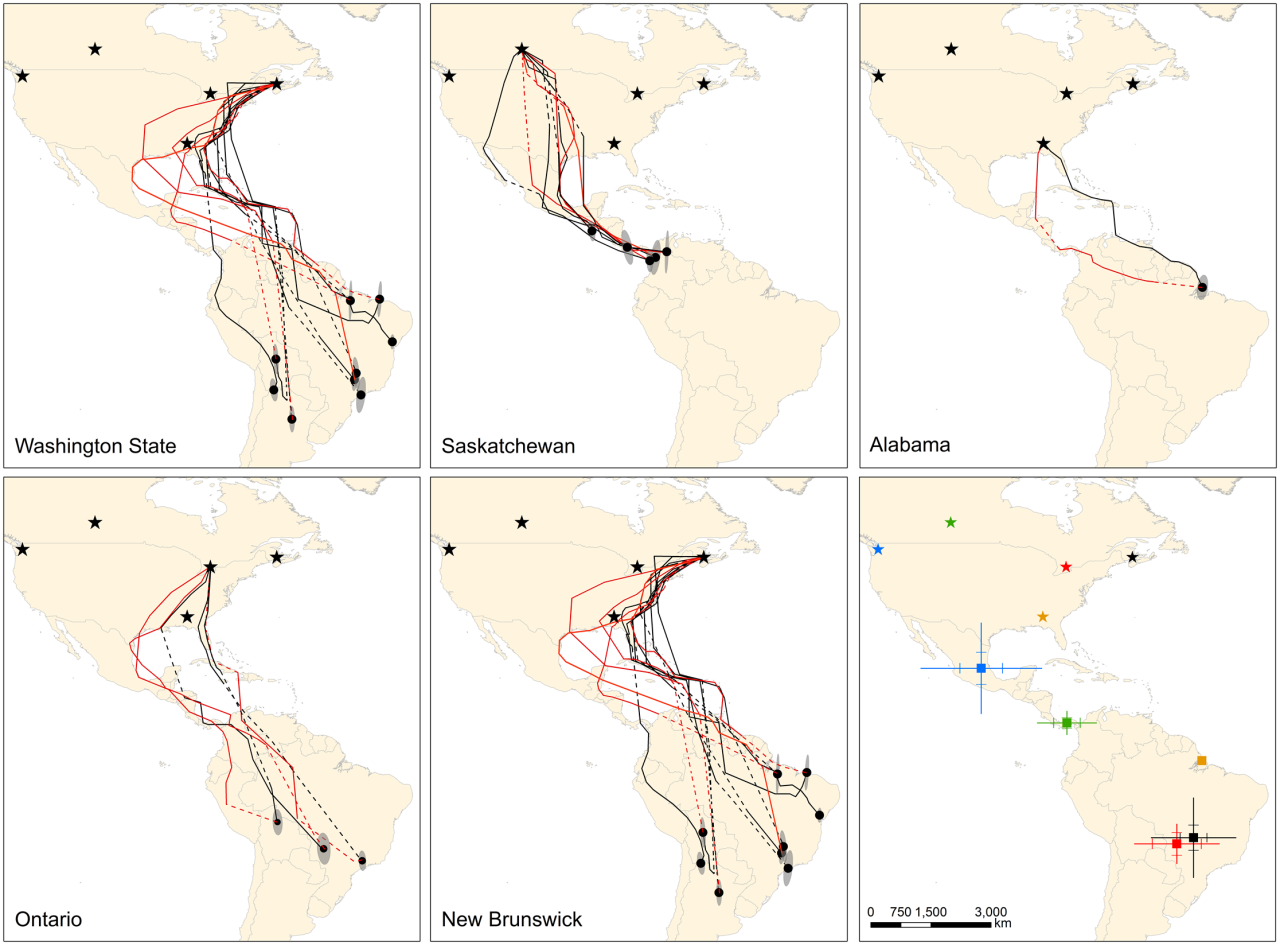
Estimated fall and spring migration routes and wintering sites for barn swallows from five populations where archival light-level geolocators were deployed. Predicted fall (black lines) and spring (red lines) migration routes and mean wintering locations (black dots) from Kalman filter state-space models. Dashed lines are estimated routes during periods when locations could not be accurately assessed (e.g., equinox, unrealistic points). Ellipses represent directional standard deviations of points used to calculate mean wintering locations. Breeding locations are denoted (stars). Data are for 13 females, 13 males and 1 unknown sex. The last panel indicates mean ± SD and SE (hash marks) for estimated mean wintering locations for five populations (blue = Washington State, green = Saskatchewan, red = Ontario, black = New Brunswick, orange = Alabama).

Departure dates from breeding grounds varied by population across years with birds from Washington State leaving significantly later ($\overset{-}{x}\;$ = September 19) than birds from other study populations except Ontario ($\overset{-}{x}\;$ = August 28; F_3, 22_ = 6.35, P < 0.005, adjusted *r* ^2^ = 0.39; Table 2 and S1 Table). Although not included in the models, the departure date (July 23) for the barn swallow from Alabama was among the earliest across all populations. Fall migration routes depended on the breeding population with swallows from Washington State (n = 8) generally following the Pacific Coast to wintering areas (Fig 1). Migration routes for Saskatchewan swallows (n = 5) were more variable with most birds migrating over the Great Plains towards Central America with one bird apparently moving southwest to the Baja California peninsula and one bird crossing the Gulf of Mexico from Louisiana to southern Mexico. Birds from the Ontario (n = 3), New Brunswick (n = 10) and Alabama (n = 1) populations typically migrated directly south or along the eastern coastal states of the United States through Florida and the Caribbean Islands towards wintering grounds. Most birds from these populations entered South America via Venezuela (n = 8) or the northeastern tip of Colombia (n = 3) with one bird crossing the Caribbean Sea from Cuba to Panama and one Ontario bird traversing the Gulf of Mexico from the Mississippi Delta to the Yucatan Peninsula. Estimated fall migration paths from breeding to wintering grounds for Washington State ($\overset{-}{x}$ = 4,805 ± 2,040 km) were significantly shorter than all other locations and routes of Saskatchewan ($\overset{-}{x}\;$ = 7,781 ± 949 km) birds were significantly shorter than routes of New Brunswick birds ($\overset{-}{x}\;$ = 10,423 ± 905 km; F_3,22_ = 23.76, P < 0.001, adjusted *r* ^2^ = 0.73). The route used by the Alabama swallow (6,198 km) was also shorter than the migration distances for the other populations except Washington State. Population was also a signification predictor of the orthodromic distance between breeding and wintering grounds with Washington State birds having shorter distances than eastern populations (F_3,22_ = 16.66, P < 0.001, adjusted *r* ^2^ = 0.65).

### Wintering Areas

Barn swallow populations showed an east-west migratory divide with Washington State and Saskatchewan birds settling farther west (F_3,22_ = 33.61, P < 0.001, adjusted *r* ^2^ = 0.80) and north (F_3,22_ = 38.06, P < 0.001, adjusted *r* ^2^ = 0.82 and MANOVA, Pillai’s trace statistic approximate F_3,22_ = 6.27, P <0.001) than birds from the two eastern populations (Fig 1). There were no significant differences between winter longitude for birds from Washington State and Saskatchewan and between birds from Ontario and New Brunswick (P ≥ 0.05). Individual wintering locations (mean and standard deviational ellipses) of birds from the Washington State population were farther north than those from Saskatchewan with mean locations in Mexico (n = 4), Guatemala (n = 1), Honduras (n = 1), Panama (n = 1) and southern Oregon (n = 1). Wintering locations for Saskatchewan swallows were in Mexico (n = 1), Panama (n = 1) and northwestern Colombia (n = 3). Ontario and New Brunswick birds had a scattered distribution of wintering areas, settling across central South America south of the Amazon basin: Ontario birds wintered in northern Bolivia (n = 1) and southern Brazil (n = 2) and New Brunswick birds wintered in eastern and southern regions of Brazil (n = 7), Argentina (n = 2) and Bolivia (n = 1). The mean winter location for the Alabama swallow was centered on the Amazon delta in Brazil and was farther north (range ~300–3,400 km) than the wintering areas of all of the Ontario and New Brunswick birds.

**Fig 2:**
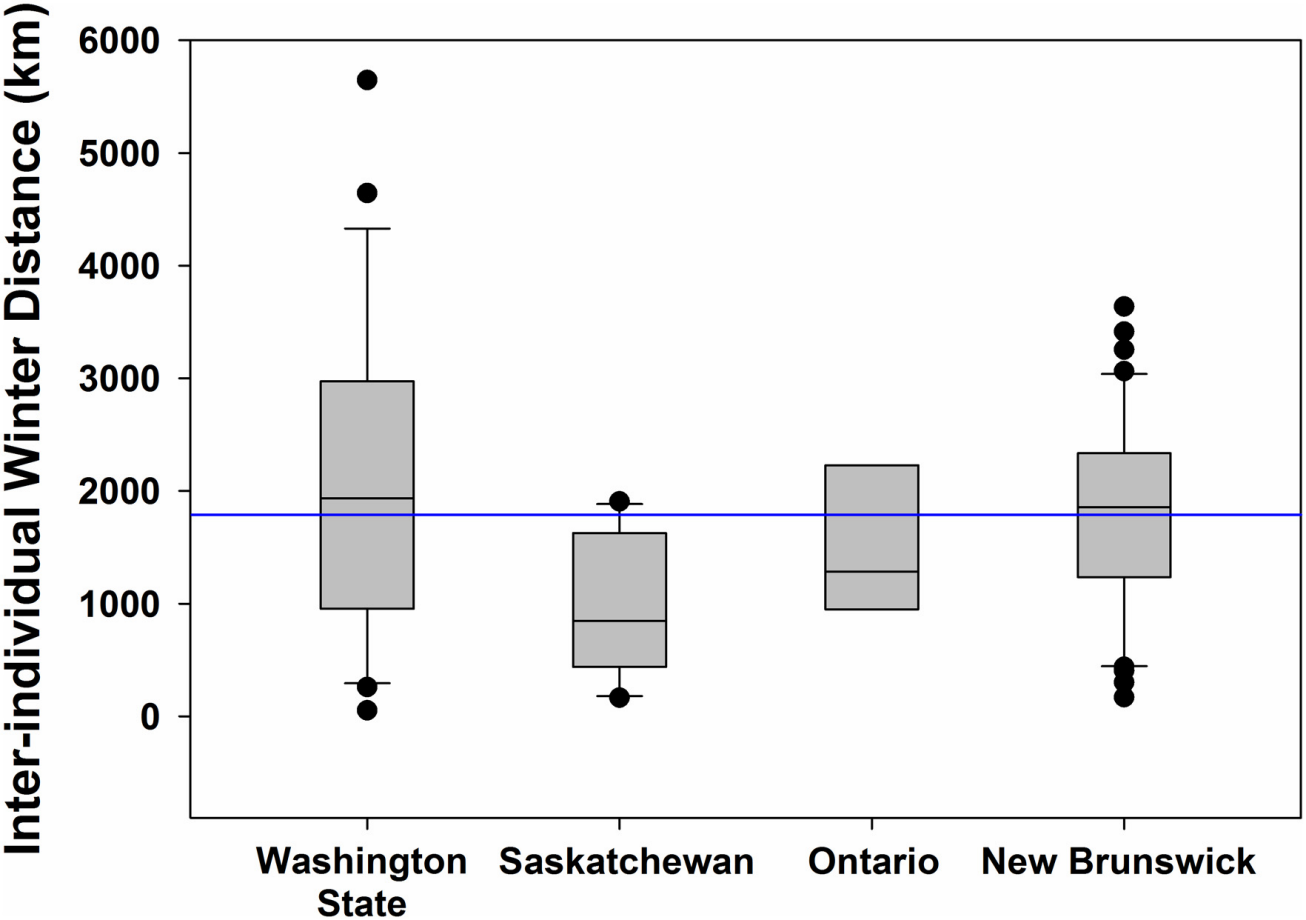
Inter-individual distances (km) between wintering sites (from mean wintering location; see Methods) of barn swallows fit with archival light-level geolocators from four populations in North America. The horizontal midlines within the boxes represent the median value, boxes depict the 25^th^ to 75^th^ percentile range of the data, the whiskers extend 1.5 times beyond the interquartile range, black circles indicate outliers and the horizontal blue line denotes the overall mean of inter-individual wintering distances (1,788 km).

Barn swallows showed low migratory connectivity with birds from Saskatchewan (orthodromic distance; $\overset{-}{x}\;$ = 948 ± 603 km) wintering closer together than birds from other populations (Figs 1 and 2). Washington State birds had a large north-south gradient in wintering areas, settling an average of 2,093 ± 1,401 km apart. Birds from Ontario and New Brunswick were dispersed across the central part of South America, settling 1,488 ± 663 km and 1,793 ± 867 km apart, respectively. Population was a significant predictor of arrival date to wintering grounds (F_3,22_ = 9.24, P < 0.001, adjusted *r* ^2^ = 0.50) but not sex (F_1,23_ = 1.66, P = 0.21, adjusted *r* ^2^ = 0.03). Saskatchewan birds ($\overset{-}{x}\;$ = November 30 ± 14.0 d) arrived significantly later to the breeding grounds than Ontario ($\overset{-}{x}\;$ = October 15 ± 20.8 d) and New Brunswick ($\overset{-}{x}\;$ = October 24 ± 14.3) birds but not Seattle ($\overset{-}{x}\;$ = November 14 ± 15.7 d) birds. Swallows from Seattle arrived on the wintering grounds significantly later than swallows from New Brunswick. Length of time at the wintering site ($\overset{-}{x}\;$ = 151 ± 19 d) was not significant for sex (F_1,18_ = 2.88, P = 0.11, adjusted *r* ^2^ = 0.09) or population (F_3,16_ = 0.74, P = 0.54, adjusted *r* ^2^ = -0.04).

### Spring Migration Timing and Routes

Departure from wintering grounds was significantly later for Saskatchewan ($\overset{-}{x}$ = April 27 ± 4.8 d) than Ontario ($\overset{-}{x}$ = March 28 ± 13.6 d) and New Brunswick ($\overset{-}{x}$ = March 24 ± 5.6) birds (F_3,16_ = .7.58, P < 0.001, adjusted *r* ^2^ = 0.51). Estimated travel distance to breeding grounds varied by population with birds from Washington State ($\overset{-}{x}\;$ = 4,528 ± 2,558 km) and Saskatchewan ($\overset{-}{x}\;$ = 7,525 ± 758 km) travelling significantly shorter distances than eastern Canadian birds (Ontario $\overset{-}{x}\;$ = 10,133 ± 604 km; New Brunswick $\overset{-}{x}\;$ = 11,287 ± 2,004 km; F_3,13_ = 12.42, P < 0.001, *r* ^2^ = 0.68). Six of 9 (67%) birds from Ontario, New Brunswick and Alabama for which geolocator data were available performed a loop pattern of migration travelling from South America towards the Yucatan Peninsula then via Mexico or directly across the Gulf of Mexico to the United States (Fig 1). Migration distances for birds that undertook this loop migration were on average 1,712 km and 2,565 km longer than their fall migration route for Ontario (n = 2) and New Brunswick (n = 3) birds, respectively, and 722 km longer for the swallow from Alabama. In contrast, estimated spring migration routes were 277 and 255 km shorter for Washington State (n = 4) and Saskatchewan (n = 4) birds, respectively. These differences meant that birds from Washington State and Saskatchewan spent significantly shorter time on migration than eastern birds (F_3,11_ = 12.15, P < 0.001, *r* ^2^ = 0.71) taking ~28 fewer days to reach their breeding grounds. Birds from Washington State arrived on breeding grounds an average of 19 days ($\overset{-}{x}$ = April 26 ± 14.8 d) earlier than other populations combined ($\overset{-}{x}$ = May 15 ± 7.6 d).

## Discussion

Our study provides, for the first time, strong evidence for a continental migratory divide for barn swallows breeding in North America. Eastern populations are clearly long-distance migrants travelling via the eastern seaboard and Caribbean Islands to central South America on fall migration whereas western populations travel to more northern wintering locations in Central America and northwestern South America via a western route. This results in later departure dates from breeding grounds for Washington State vs. other populations during the years of this study. This divide also has consequences that include differential exposure to climate cycles, weather and conditions on the wintering grounds and during migration for each migratory strategy [13]. Eastern birds are also subjected to longer migrations and consequently greater energetic demands during migration than western birds. We could not account for year effects in our data and we recognize that there may be effects of year on phenology and wintering locations [31]; however, our data show an apparent migratory divide between western and eastern breeding populations. Additional years of geolocator data from the same study populations including for individual birds would help to elucidate any potential year effects on variables impacting phenology and wintering areas (e.g., climate). In the Palearctic-Afrotropical migration system, barn swallows show a similar migratory divide whereby northern populations (British, Scandinavian and Northeast European) winter in South Africa, southern breeding populations (Switzerland, Italy, Spain) winter in Central and Eastern Africa [34–36] and Central European breeding populations show a mixed migration strategy [31, 35]. In both the New and the Old World, such migratory divides likely arose as a consequence of Pleistocene glaciations [37].

Mortality of migratory songbirds is often highest during the migration period [4, 5] and the migratory divide and subsequent routes and distances for North American barn swallows may thus contribute to differential population trajectories among regions. Swallows from the two western populations primarily migrated overland to and from their wintering grounds and avoided many of the risks that birds from the eastern populations potentially encounter while crossing the Caribbean Sea and Gulf of Mexico (e.g. storms, insufficient fat reserves). Most eastern birds also undertook a loop pattern on spring migration around the Gulf of Mexico which added thousands of kilometers to their total travel distance. Presumably, longer migration distances and times increase mortality risk through exposure to inclement weather, predation and reduced physiological condition. However, which factors are most important in influencing mortality during migration are not known and other biotic (e.g. plant and insect phenology, predation risk) and abiotic (e.g. weather, barriers, migration distance, strikes) elements likely operate concurrently to affect survival.

Despite a strong east vs. west migratory divide for barn swallows in North America, we do not observe a strong east vs. west effect in the population trend data. Most populations west of the Rocky Mountains, throughout Canada, the Great Lakes region, along the northern and central Atlantic seaboard and south through the Appalachian Mountains are declining whereas southern, north-central and U.S. Midwest populations are typically increasing or stable [13]. Unfortunately, despite five geolocator recoveries, we obtained only one functioning instrument for the stable southern population site in Alabama. That individual showed a relatively short-distance migration to northern Brazil suggesting that eastern populations of swallows may use a leapfrog migration pattern which is consistent with results from previous research on this species using stable isotopes [21]. If so, it is possible that the declining northern populations of barn swallows in eastern North America are influenced by factors operating during their long-distance migration. In a recent analysis, Michel, Smith [13] revealed that declining populations of five species of aerial insectivores, including barn swallow, were concentrated in northern North America, and were more common in long-distance than short-distance migrant species. In that analysis, they assumed all barn swallow populations were long-distance migrants but our results indicate that this species should now be considered as having a structured population that ranges from short- to long-distance migrant with some western populations (i.e. Washington State) wintering farther north than previously thought [14]. Nevertheless, Michel, Smith (13] found that population trajectories among aerial insectivores were highly idiosyncratic in space and time, suggesting the actions of numerous intrinsic and extrinsic factors driving population trends.

Here, we examined primarily northern breeding populations of barn swallows which are all showing declines and so it is not clear if migration distance per se is a key driver in regulating swallow populations. However, García-Pérez, Hobson [15] compared survivorship between barn swallows breeding at our Washington State site with that of swallows breeding at our Ontario site. They found that survivorship in the Washington State population was strongly influenced by El Niño Southern Oscillation (ENSO) whereas survivorship for the Ontario population was not. Our study provides an explanation for this result because Ontario birds apparently winter in central South America and so avoid effects of ENSO operating on the wintering grounds and potentially also on the breeding grounds. Climate cycles such as ENSO and North Atlantic Oscillation (NAO) have well-known regionally varying effects on local weather conditions and, consequently, plant productivity, arthropod abundance, and abundance, demography and energetics of migratory birds, including aerial insectivores [13, 38–40]. ENSO affects local temperature and precipitation on both the breeding and wintering grounds. During winter and spring migration, Central America, northern South America, and northern North America are warmer and drier in El Niño (ENSO-positive) years; whereas the southern half of the United States and northern Mexico are cooler and wetter [41]. The lack of an ENSO effect for the eastern (Ontario) population compared to the western (Washington State) population is entirely consistent with our finding of western birds wintering in northern Central America and where they are strongly influenced by ENSO compared to those wintering in South America. Possibly, southern, stable populations also escape ENSO effects by wintering far enough south into northern South America.

There are several potential issues regarding the use of geolocators to track movements and phenology of small migratory birds. Negative impacts of the added weight and drag of geolocators have previously been documented and associated with increased physiological stress and reduced survival [42]. Geolocators potentially affect re-fuelling rates, wintering lengths and locations, migration routes and phenology and may be exacerbated for aerial insectivores that are highly dependent on their proficiency in catching prey while in flight. These prospective impacts, along with the often large errors in location estimates and phenology (e.g. arrival and departure dates) from light-level geolocation methods, emphasize the need for cautious interpretation of results from geolocators due to the potential for biased results. However, the return rates of barn swallows fit with geolocators for our research was similar to a survivorship study at two of the same study locations where return rates of birds without geolocators were estimated [15]. Additionally, birds showed consistent patterns in migration phenology and locations between and within populations across years and with results from a previous isotope study [21] indicating that the small geolocators and the increased angle of the light logger we used do not have the same impact on barn swallows as older, larger models used in other studies [43, 44].

We used a state-space model, initially developed for fish tracking [26], to assist with analysis and interpretation of geolocator data. We acknowledge that there may be concerns regarding the use of state-space models utilizing raw geolocation estimates namely that the validity and use of the models is dependent on the quality of the raw input. However, results from our analysis using state-space models were consistent with raw positions but increased the accuracy of raw geolocations through Kalman filtering. The use of state-space models to estimate positions is well-developed in fish research [29, 30] and we advocate further development of these models for use in the study of small passerines. Nonetheless, the main results of our study that show a migratory divide in North American barn swallow populations are exceptionally clear and we are confident that an analysis using other methods would produce similar results.

## Supporting Information

S1 Table Detailed phenology data over the annual cycle for Barn Swallows fit with archival light-level geolocators from five populations, 2011–2013. (XLSX)

## References

[1] Faaborg J, Holmes RT, Anders AD, Bildstein KL, Dugger KM, Gauthreaux SA Jr., et al Recent advances in understanding migration systems of New World land birds. Ecol Monogr. 2010;80:3–48. 10.1890/09-0395.1

[2] Norris DR, Marra PP, Kyser TK, Sherry TW, Ratcliffe LM. Tropical winter habitat limits reproductive success on the temperate breeding grounds in a migratory bird. Proc R Soc B. 2004;271:59–64. 10.1098/rspb.2003.2569 . 15002772 PMC1691559

[3] Drake A, Rock CA, Quinlan SP, Martin M, Green DJ. Wind speed during migration influences the survival, timing of breeding, and productivity of a neotropical migrant, *Setophaga petechia* . PLoS ONE. 2014;9(5):e97152 Epub 2014/05/16. 10.1371/journal.pone.0097152 24828427 PMC4020938

[4] Sillett S, Holmes RT. Variation in survivorship of a migratory songbird throughout its annual cycle. J Anim Ecol. 2002;71:296–308. 10.1046/j.1365-2656.2002.00599.x

[5] Newton I. Can conditions experienced during migration limit the population levels of birds? J Ornithol. 2006;147(2):146–66. 10.1007/s10336-006-0058-4

[6] Gruebler MU, Korner-Nievergelt F, Naef-Daenzer B. Equal nonbreeding period survival in adults and juveniles of a long-distant migrant bird. Ecology and evolution. 2014;4(6):756–65. Epub 2014/04/01. 10.1002/ece3.984 24683458 PMC3967901

[7] Sherry TW, Holmes RT. Summer versus winter limitation of populations: conceptual issues and evidence In: Martin T, Finch D, editors. Ecology and management of Neotropical migratory birds: a synthesis and review of the critical issues New York, New York: Oxford University Press; 1995 p. 85–120.

[8] Webster MS, Marra PP, Haig SM, Bensch S, Holmes RT. Links between worlds: unraveling migratory connectivity. Trends Ecol Evol. 2002;17(2):76–83. 10.1016/S0169-5347(01)02380-1

[9] Nebel S, Mills A, McCracken JD, Taylor PD. Declines of aerial insectivores in North America follow a geographic gradient. Avian Conservation and Ecology. 2010;5(2):1 10.5751/ACE-00391-050201

[10] Rioux Paquette S, Garant D, Pelletier F, Bélisle M. Seasonal patterns in tree swallow prey (Diptera) abundance are affected by agricultural intensification. Ecol Appl. 2013;23(1):122–33. 10.1890/12-0068.1 23495641

[11] Hansen MC, Potapov PV, Moore R, Hancher M, Turubanova SA, Tyukavina A, et al High-resolution global maps of 21st-century forest cover change. Science. 2013;342(6160):850–3. Epub 2013/11/16. 10.1126/science.1244693 . 24233722

[12] Schreinemachers P, Tipraqsa P. Agricultural pesticides and land use intensification in high, middle and low income countries. Food Policy. 2012;37(6):616–26. 10.1016/j.foodpol.2012.06.003

[13] Michel NL, Smith AC, Clark RG, Morrissey C, Hobson KA. Continental-scale analysis of population trends of aerial insectivores reveals diverse spatiotemporal patterns. Unpublished manuscript.

[14] Brown CR, Bomberger Brown M. Barn swallow (*Hirundo rustica*), The Birds of North America Online. Ithaca: Cornell Lab of Ornithology 1999 Available: http://bna.birds.cornell.edu/bna/species/452.

[15] García-Pérez B, Hobson KA, Albrecht G, Cadman MD, Salvadori A. Influence of climate on annual survival of barn swallows (*Hirundo rustica*) breeding in North America. The Auk. 2014;131(3):351–62. 10.1642/auk-13-145.1

[16] Stutchbury BJ, Tarof SA, Done T, Gow E, Kramer PM, Tautin J, et al Tracking long-distance songbird migration by using geolocators. Science. 2009;323:896 10.1126/science.1166664 19213909

[17] Hill R. Theory of geolocation by light levels In: LeBoeuf BJ, Laws RM, editors. Elephant Seals: Population Ecology, Behaviour, and Physiology. Berkeley, CA: University of California Press; 1994 p. 227–36.

[18] Fudickar AM, Wikelski M, Partecke J. Tracking migratory songbirds: accuracy of light-level loggers (geolocators) in forest habitats. Methods Ecol Evol. 2012;3(1):47–52. 10.1111/j.2041-210X.2011.00136.x

[19] McKinnon EA, Stanley CQ, Fraser KC, MacPherson MM, Casbourn G, Marra PP, et al Estimating geolocator accuracy for a migratory songbird using live ground-truthing in tropical forest. Animal Migration. 2013;1:31–8. 10.2478/ami-2013-0001

[20] Lisovski S, Hewson CM, Klaassen RHG, Korner-Nievergelt F, Kristensen MW, Hahn S. Geolocation by light: accuracy and precision affected by environmental factors. Methods Ecol Evol. 2012;3(3):603–12. 10.1111/j.2041-210X.2012.00185.x

[21] García-Pérez B, Hobson KA. A multi-isotope (δ2H, δ13C, δ15N) approach to establishing migratory connectivity of barn swallow (*Hirundo rustica*). Ecosphere. 2014;5(2):art21 10.1890/es13-00116.1

[22] Rappole JH, Tipton AR. New harness design for attachment of radio transmitters to small passerines. J Field Ornithol. 1991;62:335–7.

[23] Naef-Daenzer B. An allometric function to fit leg-loop harnesses to terrestrial birds. J Avian Biol. 2007;38(3):404–7. 10.1111/j.2007.0908-8857.03863.x

[24] Lisovski S, Hahn S, Hodgson D. GeoLight- processing and analysing light-based geolocator data inR. Methods Ecol Evol. 2012;3(6):1055–9. 10.1111/j.2041-210X.2012.00248.x

[25] R Core Team. R: A language and environment for statistical computing. In: Team RC, editor. 3.1.1 ed Vienna, Austria: The R Foundation for Statistical Computing ISBN 3-900051-07-0; 2014 10.1016/j.jneumeth.2014.06.019

[26] Sibert JR, Musyl MK, Brill RW. Horizontal movements of bigeye tuna (*Thunnus obesus*) near Hawaii determined by Kalman filter analysis of archival tagging data. Fish Oceanogr. 2003;12(3):141–51. 10.1046/j.1365-2419.2003.00228.x

[27] Kalman RE. A new approach to linear filtering and prediction problems. Journal of Basic Engineering. 1960; 82(35–45). 10.1115/1.3662552

[28] Sibert J, Nielsen A. kftrack: An add-on package for the statistical environment R to estimate most probable track from archival tagged individuals. 2002.

[29] Nielsen A, Sibert JR. State–space model for light-based tracking of marine animals. Can J Fish Aquat Sci. 2007;64(8):1055–68. 10.1139/f07-064

[30] Jonsen ID, Basson M, Bestley S, Bravington MV, Patterson TA, Pedersen MW, et al State-space models for bio-loggers: A methodological road map. Deep Sea Research Part II: Topical Studies in Oceanography. 2013;88–89:34–46. 10.1016/j.dsr2.2012.07.008

[31] Liechti F, Scandolara C, Rubolini D, Ambrosini R, Korner-Nievergelt F, Hahn S, et al Timing of migration and residence areas during the non-breeding period of barn swallows *Hirundo rustica* in relation to sex and population. J Avian Biol. 2014;45:1–12. 10.1111/jav.00485

[32] Hobson KA, Kardynal KJ. Western veeries use an eastern shortest-distance pathway: New insights to migration routes and phenology using light-level geolocators. The Auk: Ornithological Advances. 2015;132:540–50. 10.1642/auk-14-260.1

[33] Vincenty T. Direct and inverse solutions of geodesics on the ellipsoid with application of nested equations. Survey Review. 1975;23:88–93. 10.1179/sre.1975.23.176.88

[34] Saino N, Szép T, Ambrosini R, Romano M, Møller AP. Ecological conditions during winter affect sexual selection and breeding in a migratory bird. Proc R Soc B. 2004;271:681–6. 10.1098/rspb.2003.2656 . 15209100 PMC1691647

[35] Ambrosini R, Moller AP, Saino N. A quantitative measure of migratory connectivity. J Theor Biol. 2009;257(2):203–11. Epub 2008/12/26. 10.1016/j.jtbi.2008.11.019 . 19108778

[36] Hobson KA, Møller AP, Van Wilgenburg SL. A multi-isotope (δ^13^C, δ^15^N, δ^2^H) approach to connecting European breeding and African wintering populations of barn swallow (*Hirundo rustica*). Animal Migration. 2012;1:8–22. 10.2478/ami-2012-0002

[37] Newton I. The Migration Ecology of Birds. London, UK: Academic Press; 2008.

[38] Paxton KL, Cohen EB, Paxton EH, Németh Z, Moore FR. El Niño-Southern Oscillation is linked to decreased energetic condition in long-distance migrants. PLoS ONE. 2014;9(5):e95383 Epub 2014/05/03. 10.1371/journal.pone.0095383 24788978 PMC4008376

[39] Holmgren M, Scheffer M, Ezcurra E, Gutiérrez JR, Mohren GMJ. El Niño effects on the dynamics of terrestrial ecosystems. Trends Ecol Evol. 2001;16(2):89–94. 10.1016/S0169-5347(00)02052-8 11165707

[40] Cao M, Prince SD, Small J, Goetz SJ. Remotely sensed interannual variations and trends in terrestrial net primary productivity 1981–2000. Ecosystems. 2004;7(3). 10.1007/s10021-003-0189-x

[41] Trenberth KE, Caron JM. The Southern Oscillation revisited: sea level pressures, surface temperatures, and precipitation. J Clim. 2000;13:4358–65. 10.1175/1520-0442(2000)013<4358:TSORSL>2.0.CO;2

[42] Costantini D, Moller AP. A meta-analysis of the effects of geolocator application on birds. Current Zoology. 2013;59(6):697–706.

[43] Bowlin MS, Henningsson P, Muijres FT, Vleugels RHE, Liechti F, Hedenström A. The effects of geolocator drag and weight on the flight ranges of small migrants. Methods Ecol Evol. 2010;1(4):398–402. 10.1111/j.2041-210X.2010.00043.x

[44] Scandolara C, Rubolini D, Ambrosini R, Caprioli M, Hahn S, Liechti F, et al Impact of miniaturized geolocators on barn swallow *Hirundo rustica* fitness traits. J Avian Biol. 2014;45(5):417–23. 10.1111/jav.00412

