# Correction: A Continent-Wide Migratory Divide in North American Breeding Barn Swallows (*Hirundo rustica*)

**DOI:** 10.1371/journal.pone.0133104

**Published:** 2015-07-14

**Authors:** Keith A. Hobson, Kevin J. Kardynal, Steven L. Van Wilgenburg, Gretchen Albrecht, Antonio Salvadori, Michael D. Cadman, Felix Liechti, James W. Fox

Fig 1 is incorrect. Please see the corrected Fig 1 here.

**Fig 1:**
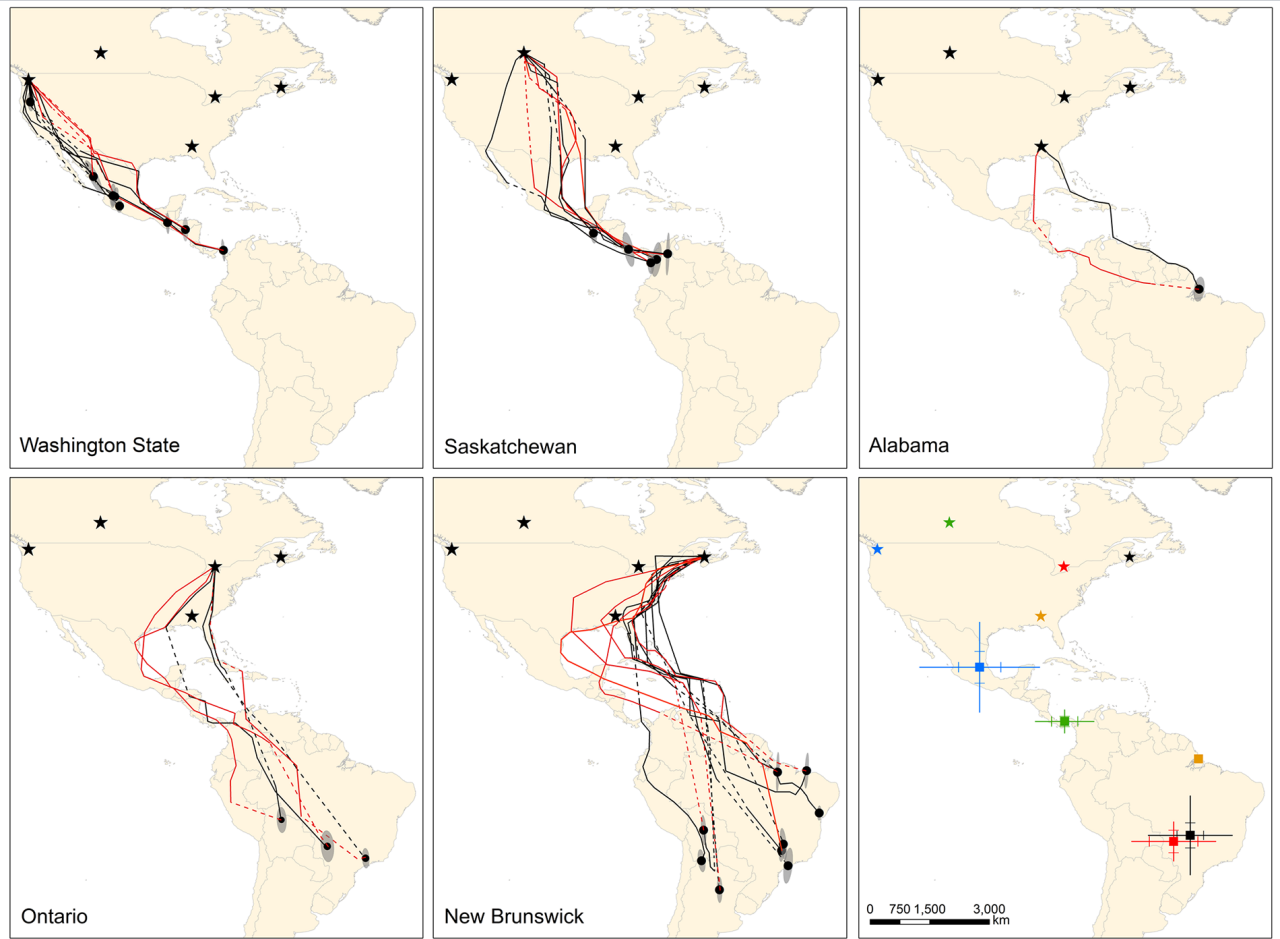
Estimated fall and spring migration routes and wintering sites for barn swallows from five populations where archival light-level geolocators were deployed. Predicted fall (black lines) and spring (red lines) migration routes and mean wintering locations (black dots) from Kalman filter state-space models. Dashed lines are estimated routes during periods when locations could not be accurately assessed (e.g., equinox, unrealistic points). Ellipses represent directional standard deviations of points used to calculate mean wintering locations. Breeding locations are denoted (stars). Data are for 13 females, 13 males and 1 unknown sex. The last panel indicates mean ± SD and SE (hash marks) for estimated mean wintering locations for five populations (blue = Washington State, green = Saskatchewan, red = Ontario, black = New Brunswick, orange = Alabama).
